# RNA-seq analysis revealed considerable genetic diversity and enabled the development of specific KASP markers for *Psathyrostachys huashanica*

**DOI:** 10.3389/fpls.2023.1166710

**Published:** 2023-03-30

**Authors:** Hao Zhang, Chunyan Zeng, Liangxi Li, Wei Zhu, Lili Xu, Yi Wang, Jian Zeng, Xing Fan, Lina Sha, Dandan Wu, Yiran Cheng, Haiqin Zhang, Guoyue Chen, Yonghong Zhou, Houyang Kang

**Affiliations:** ^1^ State Key Laboratory of Crop Gene Exploration and Utilization in Southwest China, Sichuan Agricultural University, Chengdu, Sichuan, China; ^2^ Triticeae Research Institute, Sichuan Agricultural University, Chengdu, Sichuan, China; ^3^ College of Resources, Sichuan Agricultural University, Chengdu, Sichuan, China; ^4^ College of Grassland Science and Technology, Sichuan Agricultural University, Chengdu, Sichuan, China

**Keywords:** *Psathyrostachys huashanica*, transcriptome sequencing, phylogenetic relationship, interspecific variation, KASP markers

## Abstract

*Psathyrostachys huashanica*, which grows exclusively in Huashan, China, is an important wild relative of common wheat that has many desirable traits relevant for wheat breeding. However, the poorly characterized interspecific phylogeny and genomic variations and the relative lack of species-specific molecular markers have limited the utility of *P. huashanica* as a genetic resource for enhancing wheat germplasm. In this study, we sequenced the *P. huashanica* transcriptome, resulting in 50,337,570 clean reads that were assembled into 65,617 unigenes, of which 38,428 (58.56%) matched at least one sequence in public databases. The phylogenetic analysis of *P. huashanica*, Triticeae species, and Poaceae species was conducted using 68 putative orthologous gene clusters. The data revealed the distant evolutionary relationship between *P. huashanica* and common wheat as well as the substantial diversity between the *P. huashanica* genome and the wheat D genome. By comparing the transcriptomes of *P. huashanica* and Chinese Spring, 750,759 candidate SNPs between *P. huashanica* Ns genes and their common wheat orthologs were identified. Among the 90 SNPs in the exon regions with different functional annotations, 58 (64.4%) were validated as Ns genome-specific SNPs in the common wheat background by KASP genotyping assays. Marker validation analyses indicated that six specific markers can discriminate between *P. huashanica* and the other wheat-related species. In addition, five markers are unique to *P. huashanica*, *P. juncea*, and *Leymus* species, which carry the Ns genome. The Ns genome-specific markers in a wheat background were also validated regarding their specificity and stability for detecting *P. huashanica* chromosomes in four wheat–*P. huashanica* addition lines. Four and eight SNP markers were detected in wheat–*P. huashanica* 2Ns and 7Ns addition lines, respectively, and one marker was specific to both wheat–*P. huashanica* 3Ns, 4Ns, and 7Ns addition lines. These markers developed using transcriptome data may be used to elucidate the genetic relationships among *Psathyrostachys*, *Leymus*, and other closely-related species. They may also facilitate precise introgressions and the high-throughput monitoring of *P. huashanica* exogenous chromosomes or segments in future crop breeding programs.

## 1 Introduction

The genus *Psathyrostachys* Nevski, which belongs to the tribe Triticeae, comprises eight perennial diploid or tetraploid species that contain only the Ns genome (Yen and Yang, 2011). In China, *Psathyrostachys huashanica* Keng f. ex P. C. Kuo (2*n* = 2*x* = 14, NsNs) is a nationally protected rare plant that is native to the mountainous slopes of Huashan Pass in the Qinling Mountains of Shaanxi province (Sun et al., 1993). The superior characteristics of *P. huashanica* include early maturation, tolerance to drought and salinity, and resistance to stripe rust, take-all, powdery mildew, wheat scab, and yellow dwarf. Moreover, its genome includes genes associated with many beneficial yield-related traits (Jing et al., 1999). To transfer these desirable traits into wheat, *P. huashanica* was hybridized with common wheat starting in the 1990s (Chen et al., 1991). Some progeny lines harboring *P. huashanica* chromosomal segments incorporated into the wheat genome were developed as derivative lines (Kang et al., 2009) with chromosomal additions (Kishii et al., 2010; Du et al., 2014; Tan et al., 2021), substitutions (Bai et al., 2020; Qu et al., 2022), and translocations (Li et al., 2020; Liu et al., 2021a). These progeny lines outperformed their wheat parents in terms of abiotic and biotic stress resistance and agronomic traits. To date, there has been some progress in the mapping of important genes in the *P. huashanica* genome, including those conferring resistance to stripe rust and take-all (Ma et al., 2016; Bai et al., 2021; Sun et al., 2018). Therefore, *P. huashanica* is generally considered to be a potentially useful germplasm for the genetic improvement of wheat.

Developing species-specific molecular markers that facilitate the identification of alien chromosomes or segments associated with genes of interest is critical for wheat breeding programs (Liu et al., 2018). Scholars have reported some results of genome sequencing and assembly of *P. huashanica*, while the genome data was not available at present (Li, 2019). Unfortunately, there are currently relatively few genomic and molecular marker resources for *P. huashanica*. The reported markers specific to *P. huashanica* mainly consist of expressed sequence tag-simple sequence repeats (Kanwal, 2019), sequence characterized amplified region markers, random-amplified polymorphic DNAs (Du et al., 2014) and common PCR markers (Tan et al., 2021). However, the relatively low polymorphism and distribution densities of these markers have restricted their use in wheat breeding programs and investigations of the phylogenetic relationships among *P. huashanica* and related species. Thus, additional molecular markers will need to be developed on the basis of high-throughput genotyping. Rapid advances in next-generation sequencing technologies have facilitated the large-scale identification of single nucleotide polymorphisms (SNPs) in wheat and multiple wheat-related species (Zhang et al., 2017; Ma et al., 2019). In addition, RNA sequencing (RNA-seq) technology has been used for the high-throughput and cost-effective detection of SNPs and genes as well as for analyzing phylogenetic relationships, evaluating genetic diversity, and developing molecular markers for Triticeae species (Zhou et al., 2017). To date, RNA-seq approaches have been applied to develop novel SNP markers for several wild wheat relatives, such as *Agropyron cristatum* (Zhou et al., 2017) and *Thinopyrum elongatum* (Lou et al., 2017), as well as for *Aegilops* species, including *Aegilops umbellulata* (Okada et al., 2018) and *Aegilops tauschii* (Iehisa et al., 2014). These markers were widely used for the ongoing introgression of valuable alien genes into wheat, but they also clarified phylogenetic relationships and the genetic diversity among the various genomes in Triticeae species.

We previously reported the generation of hybrids from a cross between *P. huashanica* and common wheat that did not involve an embryo rescue step and the development of some wheat–*P. huashanica* lines with useful genes for enhancing wheat characteristics (Kang et al., 2009; Kang et al., 2016). Unfortunately, the phylogenetic relationships, genetic diversity, and SNPs in *P. huashanica* and wheat remain poorly investigated. In this study, we used Illumina RNA-seq technology to generate the basal transcriptome sequencing data for *P. huashanica* and revealed genetic polymorphisms as well as phylogenetic relationships between *P. huashanica* and other Triticeae species. Furthermore, on the basis of genome-specific SNPs, we developed Kompetitive allele-specific PCR (KASP) markers for *P. huashanica*. These markers were subsequently used to elucidate the genetic differences and phylogenetic relationships among Ns, H, R, P, V, and other closely-related genomes. They were also validated regarding their utility for detecting *P. huashanica* chromosomes in wheat–*P. huashanica* 2Ns, 3Ns, 4Ns, and 7Ns addition lines.

## 2 Materials and methods

### 2.1 Plant materials

*Psathyrostachys huashanica* (2*n* = 2*x* = 14, NsNs) accession ZY3157 was collected on Huashan Mountain (Shanxi, China) by Profs. C. Yen and J. L. Yang (Sichuan Agricultural University). Wheat cultivar Chinese Spring (CS) and Chinese Spring *ph2b* (CS*ph2b*) were used as the positive controls for the molecular marker analysis and the source of blocking DNA for the Genomic *in situ* hybridization (GISH) experiments. The molecular markers were validated using the following wheat-related species: *Psathyrostachys juncea* (2*n* = 2*x* = 14, NsNs, PI314028), *Th. elongatum* (2*n* = 2*x* = 14, EE, PI531718), *Pseudoroegneria libanotica* (2*n* = 2*x* = 14, StSt, PI228391), *D. villosum* (2*n* = 2*x* = 14, VV, PI470279), *Hordeum vulgare* (2*n* = 2*x* = 14, HH, ZY11001), *A. cristatum* (2*n* = 2*x* = 14, PP, PI499389), *Secale cereale* (2*n* = 2*x* = 14, RR, QL), and *Leymus racemosus* (2*n* = 4*x* = 28, NsNsXmXm, ZY07023). Four previously identified wheat–*P. huashanica* addition lines with 2Ns, 3Ns, 4Ns, and 7Ns chromosomes were used for a KASP genotyping assay that was conducted to verify the utility of the Ns genome-specific SNPs (Zhang et al., 2022). Voucher specimens were deposited in the herbarium of the Triticeae Research Institute, Sichuan Agricultural University, China.

### 2.2 RNA−seq, transcriptome assembly and annotation

Total RNA was extracted from the flag leaves and young roots collected from five *P. huashanica* ZY3157 plants at the jointing stage using TRIzol reagent (Thermo Fisher Scientific Inc., Shanghai, China) according to the manufacturer’s instructions. The purity and concentration of the extracted RNA were then determined using the NanoDrop 2000 spectrophotometer (Thermo Fisher Scientific Inc.). The RNA integrity and quantity were determined using the Agilent 4200 system and the Agilent High Sensitivity DNA Kit (Agilent Technologies Inc., USA). The mRNA purified from the total RNA was used to construct cDNA libraries, after which the library concentrations were determined using the Qubit 3.0 fluorometer (Thermo Fisher Scientific Inc.) and by performing a qPCR assay. After preparing the sequencing libraries and pooling the libraries for different tissues, the samples were sent to the BerryGenomics Corporation (Beijing, China) for an Illumina paired-end sequencing analysis using the NovaSeq 6000 platform, which generated 150-bp paired-end reads. Reads containing adapters or more than three Ns and low-quality reads (more than 20% nucleotides with Phred quality score ≤5) were removed using an in-house Perl script to produce clean reads. The Q20, Q30, and GC content of the clean reads were calculated. The clean reads were mapped using the SILVA database and Bowtie2 (version 2.4.5) to eliminate the rRNA (Langmead and Salzberg, 2012). The Trinity program (version 2.13.2) was used for the transcriptome *de novo* assembly; the default parameters were applied, but the minimum K-mer coverage was set to 2 (Grabherr et al., 2011). The first transcript sequence generated by Trinity was selected if there were multiple isoforms. All of the unigenes identified on the basis of the Trinity assembly results were used as queries to screen the following databases using BLAST (2.11.0) (Altschul et al., 1990), with an E-value cut-off of 1e-5: NR (non-redundant protein sequences), Pfam (protein families), Swiss-Prot (manually annotated and reviewed protein sequences), eggNOG (Evolutionary Genealogy of Genes: Non-supervised Orthologous Groups), GO (Gene Ontology), and KEGG (Kyoto Encyclopedia of Genes and Genomes).

### 2.3 Phylogenetic analysis

A BLASTn search was conducted using Orthofinder (version 2.5.4) to identify the single-copy orthologous pairs between the *P. huashanica* unigenes and the coding sequences (CDSs) from *Triticum aestivum* (A, B, and D genomes were separated), *Triticum turgidum* (A and B genomes were separated), *Ae. tauschii*, *Triticum urartu*, *S. cereale*, *H. vulgare*, *Brachypodium distachyon*, *Oryza sativa*, *Zea mays*, *Sorghum bicolor*, *Setaria italica*, and *Arabidopsis thaliana* (Emms and Kelly, 2015). The CDSs were downloaded from the EnsemblPlants (https://www.plants.ensembl.org) database. The orthologous pairs that were single-copy genes in one genome and conserved in all genomes were aligned using MAFFT (version 7.508) (Katoh and Standley, 2013). The aligned genes were merged in a series for the construction of a phylogenetic tree using MEGA7 according to the neighbor-joining method (Kumar et al., 2016). The bootstrap support values were calculated with 1,000 replications and presented at each node. The maximum likelihood method was used to calculate the evolutionary distance in terms of the number of base substitutions per site, with all positions containing gaps and missing data eliminated (Tamura et al., 2004). The phylogenetic tree was drawn to scale, with branch lengths representing the evolutionary distances.

### 2.4 SNP discovery and development of KASP markers

The default parameters of the HISAT 2 software (version 2.2.1) were used to map the *P. huashanica* clean reads to the CS reference genome sequence (IWGSC RefSeqv1.0) and generate bam files (Kim et al., 2019). Picard-tools (version 1.92) and Samtools (version 1.15.1) were used to sort and mark duplicated reads as well as reorder the bam alignment results (Li et al., 2009). The SNPs and insertions/deletions (indels) were identified using Samtools, with the minimum mapping quality set to 1 and the filtering flag set to 0.002 (Li et al., 2009). Variations were annotated using Annovar on the basis of their genomic locations and filtered (total coverage >4 and quality >30) to obtain high-confidence variations (Yang and Wang, 2018). The distribution of SNPs and indels on *P. huashanica* and common wheat chromosomes was visualized using the Circos software (0.69.8) (Krzywinski et al., 2009). Homozygous SNPs and contextual sequences that did not overlap the intron in the wheat genome were analyzed using the online platform Polymarker (https://www.polymarker.info) with 100-bp flanking sequences on each side to generate KASP primers. All primers were synthesized by Sangon Biological Engineering Co., Ltd. (Shanghai, China). Details regarding the primers are presented in **Supplementary Table S1**.

### 2.5 KASP marker validation and sequential FISH and GISH analyses

The specificity, stability, and universality of the KASP markers were assessed using five individual plants of *P. huashanica* ZY3157, CS, CS*ph2b*, wheat–*P. huashanica* addition lines (2Ns, 3Ns, 4Ns, and 7Ns), and 14 wheat-related species, including *P. juncea*, *L. racemosus*, *Th. elongatum*, *Pse. libanotica*, *D. villosum*, *H. vulgare*, *A. cristatum*, and *S. cereale*. The KASP genotyping and fluorescence data analysis were performed as previously described (Ma et al., 2019). The slides for the mitotic metaphase chromosomes were prepared according to a published method (Han et al., 2006). The FISH assay involving oligonucleotide probes and the GISH assay involving *P. huashanica* DNA labeled with fluorescent tags (nick translation method of the Atto550 NT labeling kit; Jena Bioscience, Jena, Germany) were completed using the mitotic chromosomes of the wheat–*P. huashanica* addition lines to identify the added *P. huashanica* chromosomes. For the GISH assay, CS or CS*ph2b* genomic DNA was used for blocking. The FISH and GISH protocols were described in our previous report (Zhang et al., 2022). The samples on slides were counterstained with a 4,6-diamino-2-phenylindole solution (Vector Laboratories, Burlingame, CA, USA) and then examined using the BX-63 microscope (Olympus, Tokyo, Japan). Images were captured using the DP80 CCD camera (Olympus) installed on the microscope.

## 3 Results

### 3.1 Analysis of the *P. huashanica* transcriptome data and annotation of unigenes

The sequencing of the *P. huashanica* transcriptome generated 53,288,309 raw reads, of which 50,337,570 were clean reads, with a GC content of 55.75% as well as Q20 and Q30 scores of 97.01% and 92.83%, respectively (**Table 1**). The *de novo* assembly using the high-quality filtered reads produced 264,519 transcripts and 65,617 unigenes. The unigene lengths ranged from 306 to 11,879 bp, with a mean length of 1,208 bp and an N50 of 1,879 bp (**Table 2**; **Supplementary Figure S1**).

**Table 1:** Summary of the RNA_Seq data.

Raw reads	Clean reads	Clean reads/raw reads (%)	Q20 (%)	Q30 (%)	Average GC content (%)
53,288,309	50,337,570	94.46	97.01	92.83	55.75

**Table 2:** Summary of the *P. huashanica* transcriptome assembly.

Assembly parameters	
Number of transcripts assemed	264,519
Number of unigene	65,617
Maximum unigene length (bp)	11,879
Minimum unigene length (bp)	306
Average unigene length (bp)	1208
N50 length of unigene (bp)	1879

**Figure 1:**
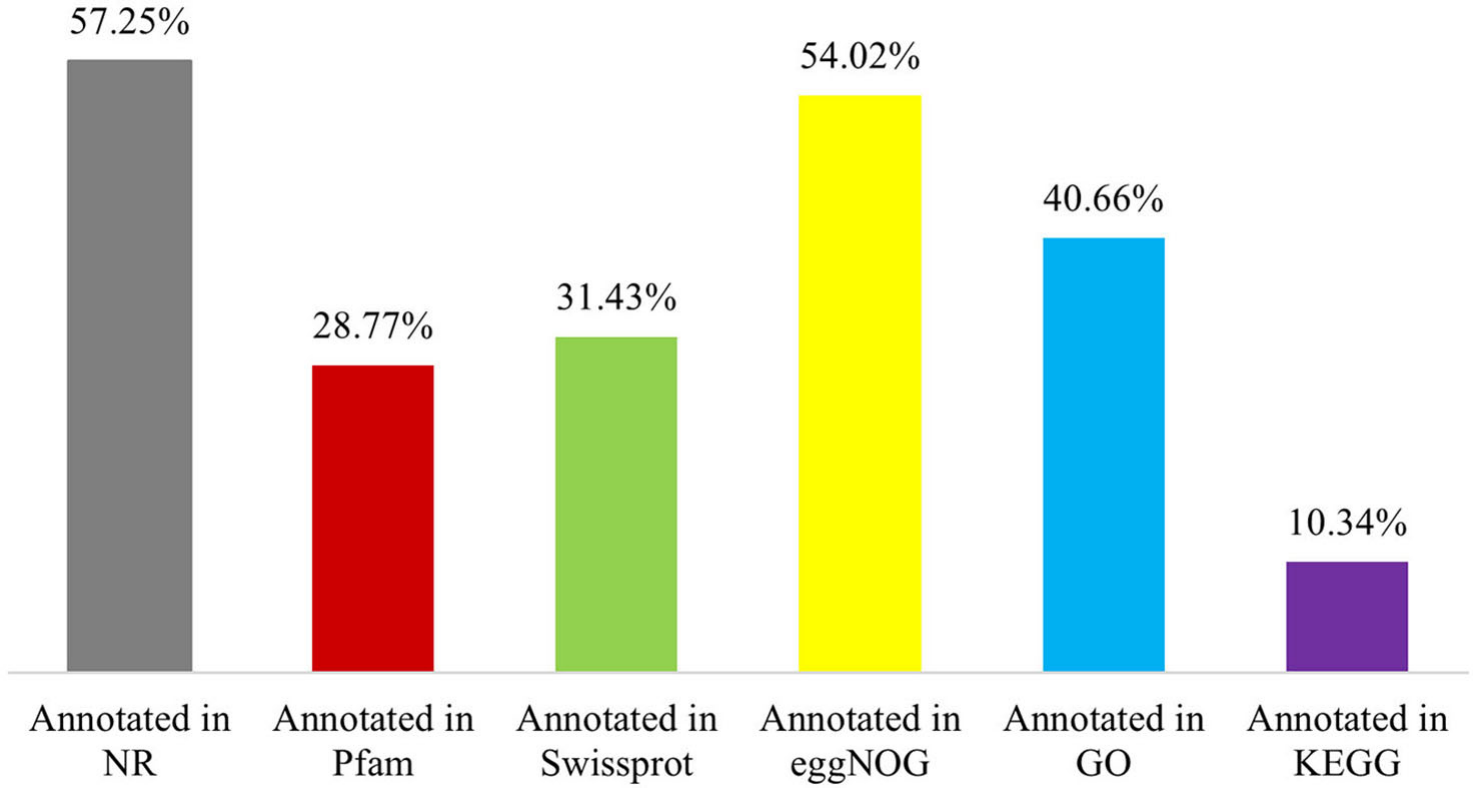
Function annotation of *P. huashanica* unigenes.

The 65,617 *P. huashanica* unigenes were annotated on the basis of six publicly available databases. More specifically, 57.25%, 28.77%, 31.43%, 54.02%, 40.66%, and 10.34% of the unigenes were annotated according to the NR, Pfam, Swissprot, eggNOG, GO, and KEGG databases, respectively (**Figure 1**; **Supplementary Table S2**). During the BLAST search, 38,428 unigenes (58.56%) had a match in at least one database (**Supplementary Table S2**). The NR database had more matches with the *P. huashanica* unigenes than the other databases. In addition, 20.94% of the unigenes were highly similar to sequences in the NR database (95%–100% sequence identity), but most of the annotated unigenes had sequence identities ranging from 80% to 95% (**Supplementary Figure S2**).

### 3.2 Identification of single-copy orthologous genes and analysis of phylogenetic relationships

**Figure 2:**
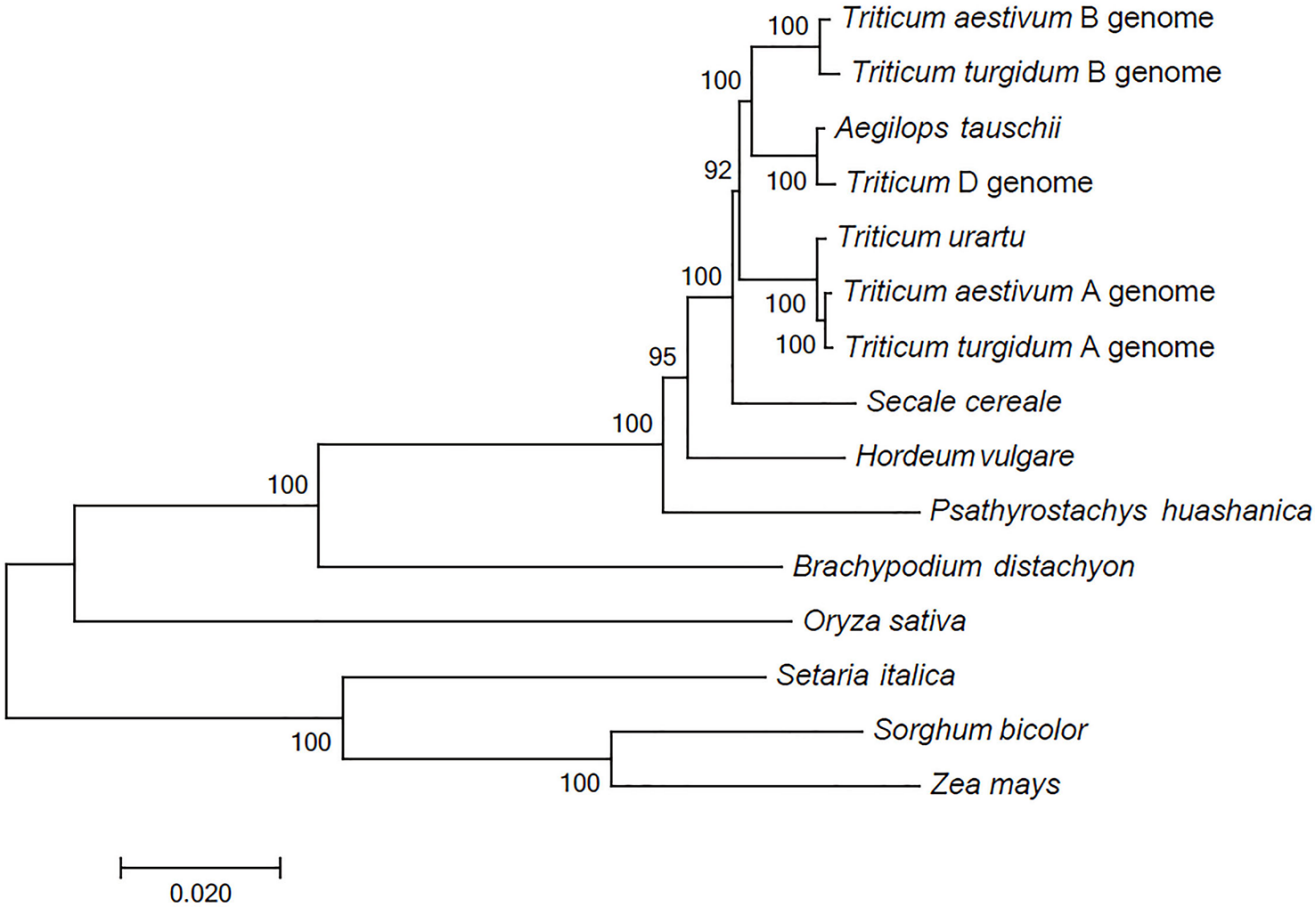
Phylogenetic relationships between *P. huashanica* and other genomes. Numbers at the node are the bootstrap values, shown as percentages. The branch lengths are the same as the evolutionary distances.

The comparison between the *P. huashanica* unigenes and the CDSs in other Poaceae and Triticeae species revealed a total of 68 putative orthologous gene clusters comprising single-copy genes that were conversed in all genomes. These putative orthologous genes were used to construct a phylogenetic tree. The phylogenetic analysis indicated that all Triticeae members were clustered into a sister clade and were closely related to *B. distachyon*, whereas the gramineous species, including *O. sativa*, *S. italica*, *S. bicolor*, and *Z. mays*, were divided into other clades. Notably, the species with the Ns genome had a more distant evolutionary relationship with *T. aestivum* and their ancestral species, including *T. urartu*, *Ae. tauschii*, and *T. turgidum*, than with *H. vulgare* and *S. cereale* (**Figure 2**). These results were consistent with the difficulties associated with the hybridization between wheat and *P. huashanica* and also suggested that the Ns genome likely contains desirable genetic variations that may be beneficial for wheat genetic studies and breeding. Moreover, the Ns genome was more distantly related to the wheat D genome than to the other wheat genomes (**Figure 2**).

### 3.3 Comparison of *P. huashanica* and *T. aestivum* sequences

**Figure 3:**
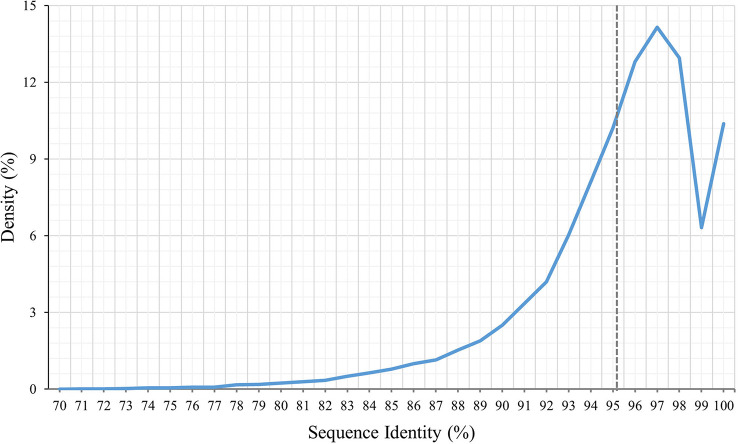
Density distribution of *P. huashanica*. Unigene sequence identity compared to the wheat.

To identify the variations between *P. huashanica* and *T. aestivum*, the transcript sequences were compared with the published sequences in the wheat CDS database. According to the BLASTn search, the average transcript sequence identity between wheat and *P. huashanica* was 95.13%, with 97.50% revealed as the most common sequence identity (**Figure 3**). Accordingly, the transcript sequences were relatively conserved between *P. huashanica* and wheat.

### 3.4 Identification of the variants between *P. huashanica* and *T. aestivum* and analysis of their effects

**Figure 4:**
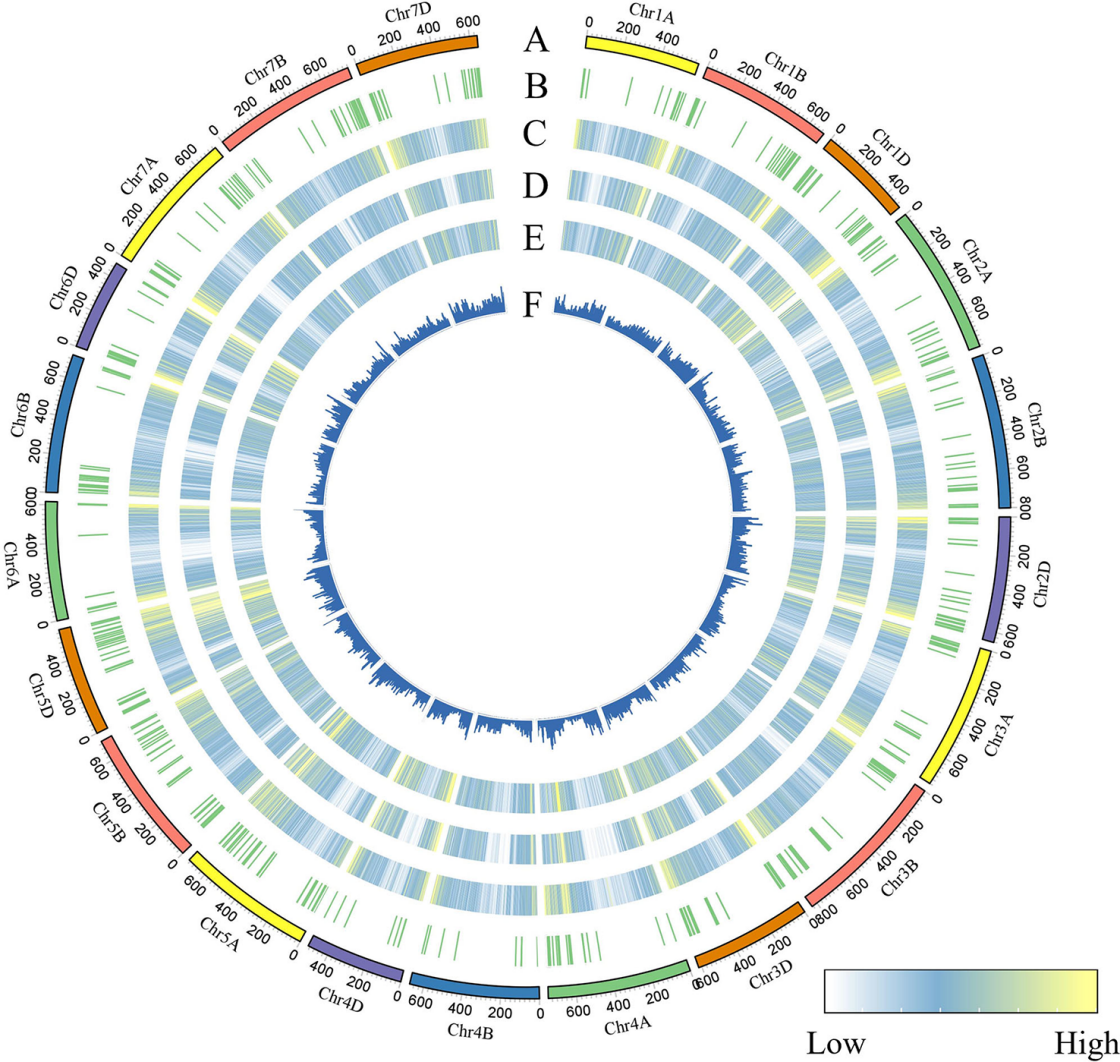
CIRCOS visualization of different data at the wheat genome-wide level. **(A)** Karyotype of the wheat genome. **(B)** Location of variant genes related to disease resistance. **(C)** Gene content density distribution; Gene density was calculated in a 5-Mb window. **(D)** Variant transcriptional density of *P.huashanica*. Variant density was calculated in transcript regions at 7-Mb window intervals. (EF) Variant density in transcript regions of *P.huashanica*. Variant density in transcript regions was visualized by heatmap **(E)** and bar **(F)** and calculated at 7-Mb window intervals.

**Figure 5:**
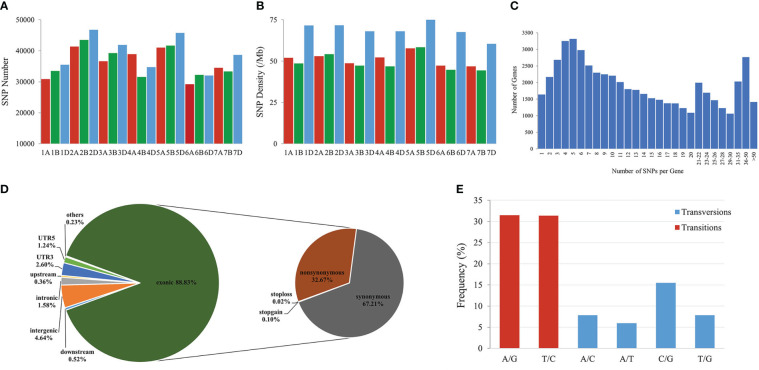
Features of SNPs between *P. huashanica* and wheat. **(A)** Distribution of SNPs in the wheat chromosomes. **(B)** Density distribution of SNPs in the wheat chromosomes. **(C)** Distribution of the number of SNPs per gene. **(D)** Distribution of SNPs in different genomic regions. **(E)** Frequency of different substitution types in the identified SNPs.

To identify genomic variants, the clean *P. huashanica* transcriptome sequencing reads were mapped to the CS reference genome sequence (IWGSC RefSeqv1.0) (**Figure 4A**). The average gene density was calculated for the wheat chromosomes. Additionally, the average gene density and variant density increased from the centromeres to the telomeres of the chromosomes (**Figures 4C–F**). Moreover, 750,759 SNPs and 3,883 indels were identified between the *P. huashanica* and CS transcripts (**Figures 4E, F**; **5A**). The variants were spread across the wheat genome, but they were not directly proportional to the chromosome length and gene number (**Figures 5A, B**). An average of 55.67 SNPs/Mb were detected in the wheat genome (**Figure 5B**). The SNP density was highest in the D genome (69.71/Mb), followed by the A genome (51.17/Mb) and the B genome (49.24/Mb) (**Figure 5B**). Furthermore, the highest and lowest SNP densities were observed for the homologous group 5 chromosomes (64.59/Mb) and the homologous group 7 chromosomes (50.11/Mb), respectively (**Figure 5B**).

The mapping of all SNPs to the wheat genes indicated 54,277 of the 107,891 annotated genes (50.31%) contained one or more SNPs, of which 668 were related to disease resistance (**Figures 4B**; **5C**; **Supplementary Table S3**). There was an average of 13.83 SNPs per gene and 25.16% of the genes had more than 20 SNPs (**Figure 5C**). Of these genes, 1,416 genes had more than 50 substitutions in the identified SNPs, whereas 9,743 genes had fewer than five substitutions (**Figure 5C**). These 1,416 genes may be highly diverse, making them potentially useful for exploring the genetic diversity among Triticeae species and for breeding novel varieties.

The distribution of the SNPs in various wheat genomic regions was determined (**Figure 5D**). A total of 12,530 (1.58%), 36,690 (4.64%), 2,811 (0.36%), and 4,108 (0.52%) variants were detected in the intronic, intergenic, upstream, and downstream regions, respectively (**Figure 5D**). Approximately 30,387 SNPs (3.84%) were located in the 5′- or 3′-UTRs, whereas 88.83% of the variants were present in the coding regions, in which the non-synonymous-to-synonymous variants ratio was 48.61% (**Figure 5D**). These results implied that the transcribed regions were likely under purifying selection. In terms of the substitution types, transitions (62.80%; A/G: 31.45% and T/C: 31.35%) were more common than transversions (37.19%; A/C: 7.87%, A/T: 5.96%, C/G: 15.5%, and T/G: 7.86%) (**Figure 5E**). The proportions of the A/G and T/C transitions were similar (i.e., 31.45% and 31.35%, respectively) (**Figure 5E**). Of the transversions, C/G was the most common (15.5%), followed by A/C and T/G (7.87%) and A/T (5.96%) (**Figure 5E**). The transitions-to-transversions ratio was 1.69:1, which reflected the genetic conservation during evolution.

### 3.5 Identification of SNPs on the basis of KASP genotyping results

**Figure 6:**
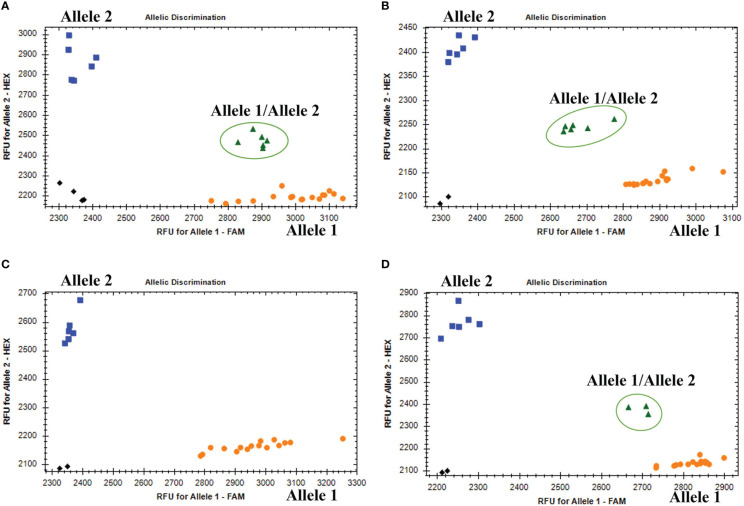
**(A)** Amplification results of marker KP2D-180993989 in CS, CS*ph2b*, wheat- *P. huashania* 3Ns, 4Ns, 7Ns addition lines (Allele 1); *P. huashanica* (Allele 2); wheat-*P. huasahnica* 2Ns addition lines (Allele 1/Allele 2). **(B)** Amplification results of marker KP7D-437429145 in CS, CS*ph2b*, wheat- *P. huashania* 2Ns, 3Ns, 4Ns addition lines (Allele 1); *P. huashanica* (Allele 2); wheat-*P. huasahnica* 7Ns addition lines (Allele 1/Allele 2). **(C)** Amplification results of marker KP2A-719278641 in *P. juncea*, *Th. elongatum*, *A cristatum*, *D villosum*, *H vulgare*, *S. cereale*, *Pse. libanotica*, *Ley. racemosus* (Allele 1); *P. haushancia* (Allele 2). **(D)** Amplification results of marker KP1A-440448839 in *Th. elongatum*, *A cristatum*, *D villosum*, *H vulgare*, *S. cereale*, *Pse. libanotica* (Allele 1); *P. haushancia*, *P. juncea* (Allele 2); *Ley. racemosus* (Allele 1/Allele 2).

The KASP genotyping assays validated 90 candidate SNPs between the *P. huashanica* and CS genomes (**Supplementary Table S1**). The allele-specific primers uncovered single nucleotide substitutions between *P. huashanica* and the other accessions. In the KASP genotyping assays, 58 primers identified obvious clusters and detected allele 2 in *P. huashanica*, but allele 1 in CS and CS*ph2b*. Hence, these SNPs, which represented 64.4% of the developed markers, were validated as specific for *P. huashanica* in a common wheat background (**Supplementary Table S1**). Among these specific markers, four and eight markers detected heterozygous alleles (allele 1/allele 2) in the wheat–*P. huashanica* 2Ns and 7Ns addition lines, respectively, whereas one marker simultaneously detected heterozygosity (allele 1/allele 2) in both wheat–*P. huashanica* 3Ns, 4Ns, and 7Ns addition lines (**Figures 6A, B**; **Supplementary Table S1**). The chromosomal compositions in these addition lines were confirmed by conducting FISH and GISH assays (**Supplementary Figure S3**). Thus, these markers were useful for detecting the corresponding *P. huashanica* chromosomes in a wheat background.

To evaluate the specificity and stability of the 58 P*. huashanica*-specific markers, the KASP genotyping assay was performed using eight wheat-related species that differed regarding their basic genome. Six of these markers detected allele 2 in *P. huashanica* while detected allele 1 or no amplification in all other wheat-related species (**Figure 6C**; **Supplementary Table S1**). In contrast, three markers detected allele 2 in both *P. huashanica* and *P. juncea*, but detected allele 1 or no amplification in all other analyzed species. Therefore, they were specific to the Ns genome in the genus *Psathyrostachys*. Five markers were specific to the Ns genome, of which two detected allele 2 in Ns genome-containing species, but allele 1 in the other species. In contrast, three markers detected allele 2 in *P. huashanica* and *P. juncea*, but allele 1/allele 2 (heterozygosity) in *L. racemosus* (**Figure 6D**; **Supplementary Table S1**).

## 4 Discussion

### 4.1 Powerful method for exploring *P. huashanica* genomic polymorphisms and developing markers

*Psathyrostachys huashanica* genes are potentially useful for increasing wheat resistance to biotic and abiotic stresses. Hence, *P. huashanica* has been used in wheat breeding programs for a long time. However, *P. huashanica* has primarily been used for molecular cytogenetic research, which resulted in the production and identification of wheat–*P. huashanica* introgression lines as well as the development of genome-specific markers, while the available genetic diversity and polymorphisms in the transcribed regions of the genome remained largely underexploited (Bai et al., 2020; Li et al., 2020; Liu et al., 2021a; Qu et al., 2022). To further explore the utility of *P. huashanica* for improving wheat, its genetic diversity should be elucidated at the molecular level and additional genetic markers should be developed.

Reference genomes are not widely available for most wild relatives of wheat because of their substantial abundance of repetitive sequences and their complexity. Although some statistics of the *P. huashanica* genome assembly have been reported, the genome resource has not been released (Li, 2019). Advances in next-generation sequencing technologies (e.g., Illumina RNA-seq) have provided researchers with alternative approaches for studying global transcriptome profiles for species lacking reference genomes. Moreover, RNA-seq-based methods are generally unaffected by the repetitiveness of non-transcribed regions and genome complexity for analyzing the transcripts of wheat relatives, evaluating genetic diversity, and identifying novel molecular markers (Zeng et al., 2017; Zhou et al., 2017). For example, Okada et al. (2018) performed an RNA-seq analysis of 12 representative *Ae. umbellulata* accessions. Many SNPs and indels were called and anchored to the pseudomolecules of *Ae. tauschii* and barley, revealing the greater genetic diversity in *Ae. umbellulata* than in *Ae. tauschii*. Zhou et al. (2017) conducted a transcriptome sequencing study to explore the genetic relationships between *A. cristatum* and wheat and wheat relatives as well as to identify the variations between *A. cristatum* and wheat. Potential SNPs were detected, of which 53 were validated according to a KASP genotyping assay. In another study, transcriptome data were used to develop 134 *Ae. longissima-*specific PCR markers, which may enable the transfer of desirable *Ae. longissima* genes into wheat *via* marker-assisted selection (Wang et al., 2018). Kanwal (2019) characterized 11 polymorphic EST-SSR primers to reveal the population genetic diversity among 12 P*. huahsancia* accessions by transcriptome analysis.

To detect genome-wide polymorphisms and develop novel genome-specific SNP markers, we conducted an RNA-seq analysis of *P. huashanica* leaf and root tissues using the Illumina NovaSeq 6000 platform, which generated 150-bp paired-end reads. The comparative analysis of *P. huashanica* and wheat transcripts indicated the average sequence identity was 95.13%, whereas the peak sequence identity was 97.6%. These findings reflect the relatively close genetic relationship between *P. huashanica* and wheat, implying molecular markers may be developed on the basis of the wheat reference genome. By mapping the transcriptome sequencing data to the wheat reference genome, many high-quality SNPs on 21 wheat chromosomes were called, with 50.31% of the predicted wheat genes containing one or more variants, indicative of a great genetic diversity in *P. huashanica*. Using the called SNPs enabled the development of genus-specific markers. Compared with the other sequencing approaches used in earlier molecular investigations, such as genotyping-by-sequencing and specific-locus amplified fragment sequencing (Elshire et al., 2011; Poland et al., 2012; Kantarski et al., 2016; Song et al., 2020), RNA-seq is a relatively cost-effective method for producing transcription data-based markers closely linked to genes associated with useful agronomic traits.

### 4.2 Phylogenetic relationships between *P. huashanica* and wheat as well as wheat relatives

Phylogenetic relationships are useful for further characterizing crops and for selecting varieties in wheat breeding programs. Although wheat–*P. huashanica* progeny lines have long been used as sources of genes related to value-added traits, the genetic relationships between *P. huashanica* and other Triticeae species remain unclear. Our phylogenetic analysis showed that *P. huashancia* has closer genetic relationship with barley and rye than with wheat. These findings are consistent with the results of similar phylogenetic analyses involving trnL-F sequences and the chloroplast genome (Chen et al., 2020) as well as the SNP validation results in this study. According to the analysis of the Ns chromosome markers in wheat-related species, 28 of the 58 specific SNP markers (48.28%) occupied similar positions in *P. huashanica* (Ns) and *A. cristatum*. The next highest percentages were observed for the comparisons with *S. cereale* (R, 46.55%), *Pse. libanotica* (St, 43.10%) and *H. vulgare* (H, 31.03%). In contrast, only 27.59% of the SNP markers occupied similar positions in *P. huashanica* (Ns), *D. villosum* and *Th. elongatum*. Thus, *P. huashanica* has a closer genetic relationship with *A. cristatum*, *S. cereale*, *Pse. libanotica* and *H. vulgare*, than with *D. villosum* and *Th. elongatum*. Notably, 47 and 43 of the specific SNP markers detected allele 2 in *P. juncea* and allele 2 or heterozygosity in *L. racemosus*, respectively. These observations provide new evidence that the genera *Leymus* and *Psathyrostachys* are genetically closely related and that *Leymus* species contain the Ns genome from *Psathyrostachys*, which is in accordance with the results of previous research (Wang and Lu, 2014; Sha et al., 2017). In addition, the SNP density was obviously higher in the wheat D genome than in the A and B genomes, suggesting that *P. huashanica* is more distantly related to wheat relatives with the D genome than to wheat relatives containing the A and B genomes. This is also supported by the phylogenetic analysis involving Ns and the wheat A, B, and D genomes. Moreover, the average gene density and variant transcriptional density increased from the centromeres to the telomeres of the *P. huashanica* chromosomes, indicating that the frequency of allelic variations was greater for the telomeres than for the centromeres. Earlier studies demonstrated that the likelihood of genetic changes (e.g., exchange, recombination, and elimination) in chromosomal regions increases as the distance from the centromere increases (Jiang et al., 1994; Fan et al., 2020), which explains the rarity of intercalary translocation lines during interspecific or intergeneric hybridizations.

### 4.3 SNP marker application

Molecular markers have been extensively used to detect and trace alien chromosomes or chromosomal segments carrying desirable genes in a wheat background to increase the selection efficiency during breeding and shorten the breeding cycle (Fedak, 1999). An increasing number of elite genes have been identified in *P. huashanica*, but the previously developed molecular tools for *P. huashanica* were relatively imprecise and inefficient (Wang et al., 2014; Kanwal, 2019). For instance, Kanwal (2019) developed a series of EST-SSR markers of *P. huashanica* based on transcriptome data. In one of our earlier studies, we developed specific PCR markers for *P. huashanica* 7Ns chromosomes, but they were not co-dominant and could not be used to simultaneously trace alien chromosomes and their homoeologous groups (Tan et al., 2021). However, KASP markers for SNP genotyping are viable alternatives that are increasingly becoming the preferred markers because of their efficiency, accuracy, and ease-of-use as well as the fact they are not dependent on gel electrophoresis (Khera et al., 2013; Semagn et al., 2014). Therefore, KASP markers have been commonly used for mapping genes, identifying alien introgression lines, and marker-assisted selection-based wheat breeding. For example, Ma et al. (2019) quickly and reliably characterized two wheat–*A. cristatum* introgression lines with increased grain numbers per spike and resistance to powdery mildew by completing a KASP genotyping assay involving 6P-specific SNP markers. Diagnostic KASP markers have been developed to trace functional genes relevant for breeding, including the leaf rust resistance gene *Lr42* (Liu et al., 2021b), the Fusarium head blight resistance gene *Fhb1* (Su et al., 2018), and putative pre-harvest sprouting resistance genes (Liu et al., 2022). In the current study, we successfully developed the specific KASP markers for *P. huashanica* and Ns genome-containing species by RNA-seq. The markers described herein may be exploited to identify *P. huashanica* chromosomes in a wheat background. Moreover, these KASP markers and SNPs potentially useful for designing KASP markers may be applicable for monitoring wheat–*P. huashanica* cryptic small alien segment introgressions, while also facilitating the marker-assisted transfer of desirable traits from *P. huashanica* into adapted wheat cultivars in breeding programs. They can also further clarify the phylogenetic and functional relationships among *Psathyrostachys* and *Leymus* species.

## Data availability statement

Raw data were deposited in NCBI SRA database. (https://www.ncbi.nlm.nih.gov/sra/?term=PRJNA937391).

## Author contributions

HZ, CZ, LL and HK conducted the experiment, analyzed the data, and drafted the manuscript. WZ, LX, YW and JZ characterized addition lines. XF, LS, HQZ, DW, YC and GC provided technique guidance for bioinformatics analysis. YZ and HK designed the experiment and formulated the questions. All authors contributed to the article and approved the submitted version.
