# Supplementary figures and images for: RNA-seq analysis revealed considerable genetic diversity and enabled the development of specific KASP markers for Psathyrostachys huashanica
Source: Front Plant Sci. 2023 Mar 30;14:1166710. doi: 10.3389/fpls.2023.1166710 (PMC10097992; doi:10.3389/fpls.2023.1166710)

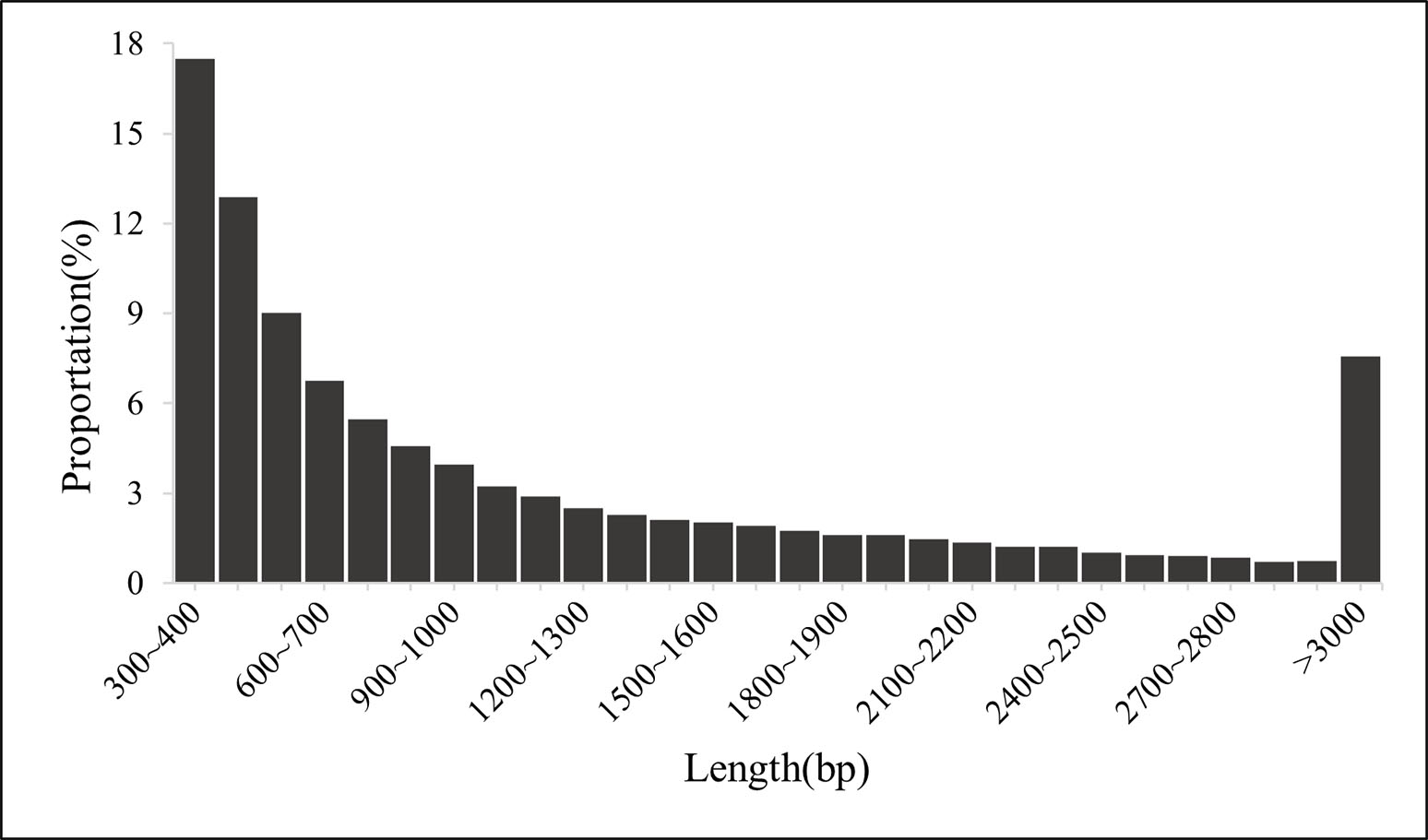

Supplement: Supplementary file 1 [file Image_1.jpeg]

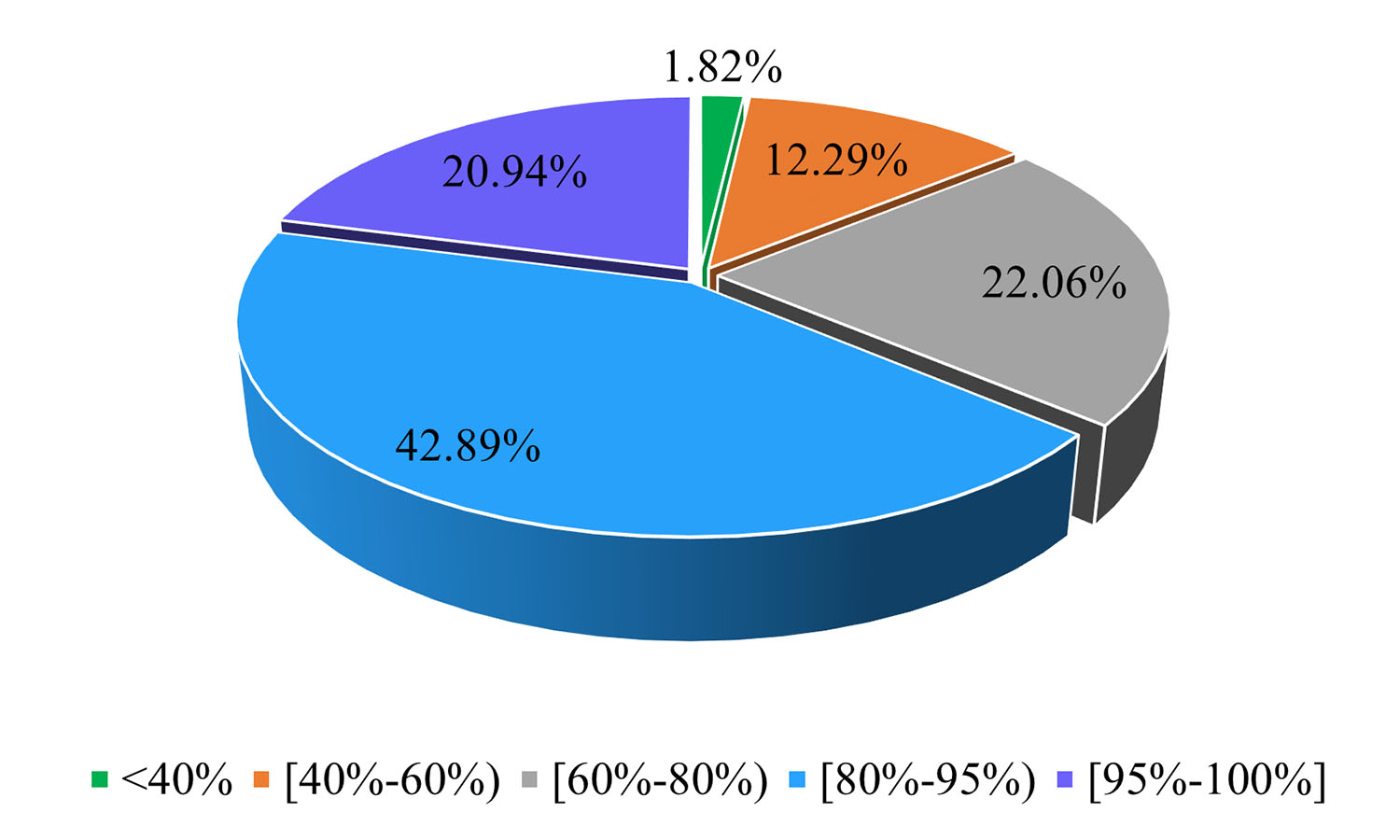

Supplement: Supplementary file 2 [file Image_2.jpeg]

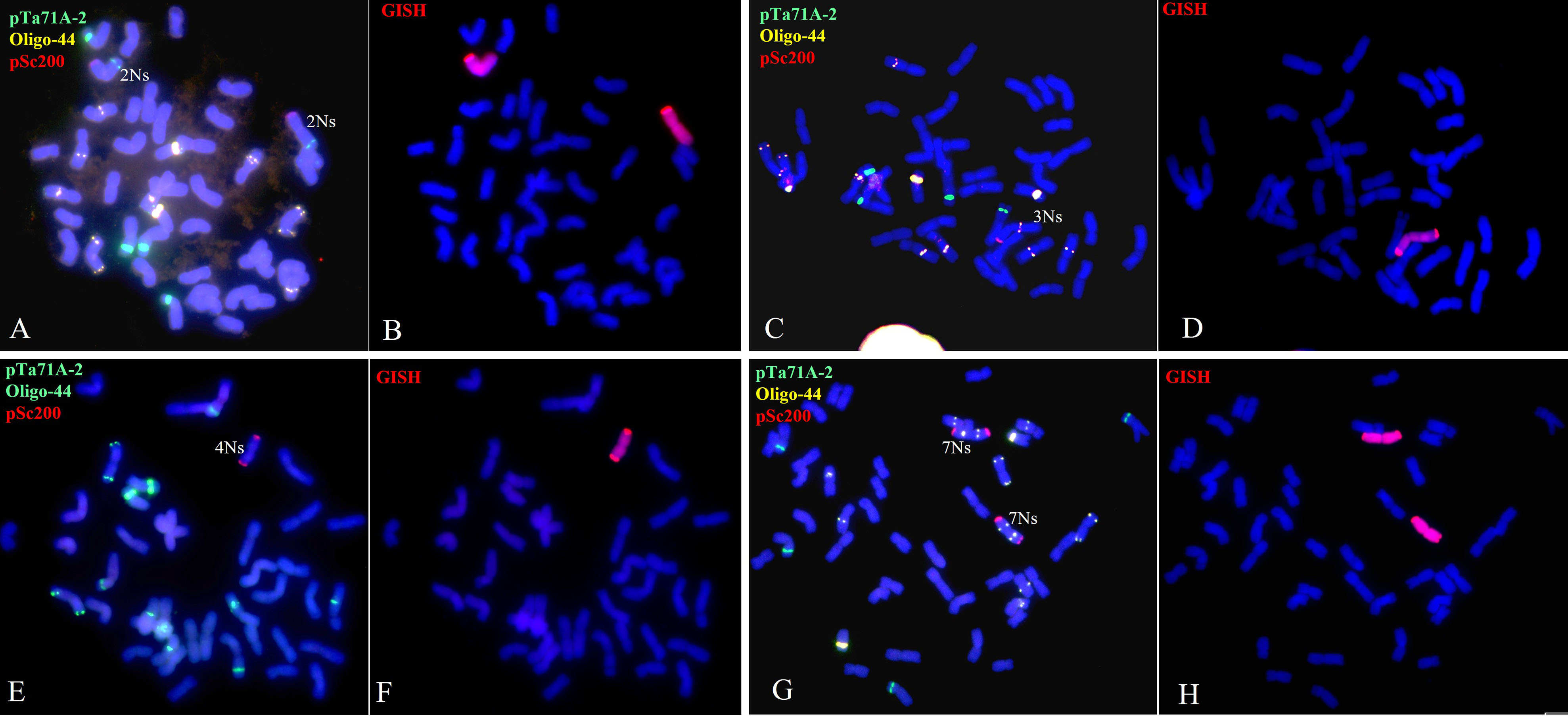

Supplement: Supplementary file 3 [file Image_3.jpeg]
